# Dynamic Changes in Circulating Endocrine FGF19 Subfamily and Fetuin-A in Response to Intralipid and Insulin Infusions in Healthy and PCOS Women

**DOI:** 10.3389/fendo.2020.568500

**Published:** 2020-09-30

**Authors:** Manjunath Ramanjaneya, Milin Bensila, Ilham Bettahi, Jayakumar Jerobin, Tareq A. Samra, Myint Myint Aye, Meis Alkasem, Kodappully Sivaraman Siveen, Thozhukat Sathyapalan, Monica Skarulis, Stephen Lawrence Atkin, Abdul-Badi Abou-Samra

**Affiliations:** ^1^Qatar Metabolic Institute, Interim Translational Research Institute, Academic Health System, Hamad Medical Corporation, Doha, Qatar; ^2^Department of Academic Endocrinology, Diabetes and Metabolism, Hull York Medical School, Hull, United Kingdom; ^3^Translational Research Institute, Academic Health System, Hamad Medical Corporation, Doha, Qatar; ^4^Royal College of Surgeons in Ireland Bahrain, Busaiteen, Bahrain

**Keywords:** PCOS (polycystic ovarian syndrome), FGF (fibroblast growth factor), Fetuin A, hyperinsulimic-euglycemic clamp, FGF19, FGF21

## Abstract

**Background:** The fibroblast growth factors (FGF) 19 subfamily, also referred to as endocrine FGFs, includes FGF19, FGF21, and FGF23 are metabolic hormones involved in the regulation of glucose and lipid metabolism. Fetuin-A is a hepatokine involved in the regulation of beta-cell function and insulin resistance. Endocrine FGFs and fetuin-A are dysregulated in metabolic disorders including obesity, type 2 diabetes, non-alcoholic fatty liver disease and polycystic ovary syndrome (PCOS). Our study was designed to examine the response of endocrine FGFs and fetuin-A to an acute intralipid, insulin infusion and exercise in PCOS and healthy women.

**Subjects and Measurements:** Ten healthy and 11 PCOS subjects underwent 5-h saline infusions with a hyperinsulinemic-euglycemic clamp (HIEC) performed during the final 2 h. One week later, intralipid infusions were undertaken with a HIEC performed during the final 2 h. After an 8 week of exercise intervention the saline, intralipid, and HIEC were repeated. Plasma levels of endocrine FGFs and fetuin-A were measured.

**Results:** Baseline fetuin-A was higher in PCOS women but FGF19, FGF21, and FGF23 did not differ and were unaffected by exercise. Insulin administration elevated FGF21 in control and PCOS, suppressed FGF19 in controls, and had no effects on FGF23 and fetuin-A. Intralipid infusion suppressed FGF19 and increased FGF21. Insulin with intralipid synergistically increased FGF21 and did not have effects on lipid-mediated suppression of FGF19 in both groups.

**Conclusion:** Our study provides evidence for insulin and lipid regulation of endocrine FGFs in healthy and PCOS women, suggesting that FGF family members play a role in lipid and glucose metabolism.

**Clinical Trial Registration:** www.isrctn.org, Identifier: ISRCTN42448814.

## Introduction

PCOS is a hormonal and metabolic disorder that affects about ten percent of women in the reproductive age group. Women with PCOS are more susceptible to insulin resistance (IR), myocardial infarction, fertility problems, type 2 diabetes (T2D), and cardiovascular disorders. The fibroblast growth factor (FGF) family has 22 members that are divided into further 7 subgroups based on phylogenetic similarity. FGFs function by binding to 4 tyrosine kinase receptors called FGF receptors (FGFRs) (1). FGF19 subfamily, also referred to as endocrine FGFs, includes FGF19, FGF21, and FGF23 that circulate in the blood and act on multiple organs involved in the regulation of metabolism. Endocrine FGFs have lower affinity for FGFRs and form complexes with Klotho proteins to elicit their biological functions in target tissues. Small intestinal cells are the main source of circulating FGF19; and in rodents FGF19 reduces hepatic triglycerides and glucose levels, promotes fatty acid oxidation by inhibiting acetyl CoA carboxylase 2, and enhances insulin sensitivity (2). FGF19 expression is regulated by farnesoid X receptor; and bile acids increase the circulating levels of FGF19 (3).

FGF21 is expressed in liver, thymus, adipose tissues, pancreas, skeletal muscle, kidney, and heart (4). Administration of FGF21 reduces bodyweight in obese mice (5, 6) and promotes insulin sensitivity in diabetic mouse models, with reductions in plasma glucose, triglycerides, glucagon, and insulin levels (4). FGF23, which is mainly produced by osteocytes, is involved in the regulation of phosphate levels and vitamin D synthesis in the kidney (7). High plasma levels of FGF23 are associated with end-stage renal disease, cardiovascular diseases, and death (8, 9). Fetuin-A is a circulating glycoprotein synthesized mainly in the liver and is involved in the regulation of insulin signaling. In adipocytes, fetuin-A increases the accumulation of triacylglycerol and fatty acid uptake and enhances lipogenesis (10). Circulating fetuin-A levels are higher in obesity, T2D and non-alcoholic fatty liver disease (NAFLD) and is associated with impaired insulin sensitivity (11). Studies have shown that fetuin-A directly interacts with the insulin receptor and alters insulin signaling by disrupting insulin-stimulated phosphorylation of the insulin receptor and insulin receptor substrate-1 (12).

Previous studies have shown differences in circulating levels of endocrine FGFs (13, 14) and fetuin-A (15) in PCOS women. FsGF21 and fetuin-A were reported to be elevated and FGF19 was found to be lower in PCOS women compared to healthy controls (13–15). Acute intralipid administration elevates circulating free fatty acid (FFA) and triglycerides, decreases peripheral glucose uptake resulting in increased insulin resistance (16–18). Regular exercise improves insulin signaling in skeletal muscle, promotes insulin sensitivity, improves cardio-metabolic fitness, and overall health (19). The role of endocrine FGFs and fetuin-A in intralipid mediated insulin resistance or in enhanced insulin sensitive states induced by exercise is not fully understood. Therefore, the aim of this study was to understand the relationship between endocrine FGFs and fetuin-A response to IR induced by intralipids and the combination of intralipids and insulin in women with PCOS and healthy control women.

## Study Design and Methodology

### Ethical Approval

The study was approved by the Yorkshire and the Humber Research Ethics Committee (reference number 10/H1313/44) and The Medical Research Center at Hamad Medical Corporation (reference number RP #17180/17) all study participants gave their written informed consent before participation. Intralipid and euglycemic clamp studies were conducted in the clinical research facility at Hull and East Yorkshire Hospital.

### Study Subjects and Insulin/Intralipid Clamp Protocol

The study subjects were matched for age, weight and BMI, non- smokers, were not on any medication and had no concurrent illness. PCOS was diagnosed according to the Rotterdam criteria (20). Eleven PCOS and 10 healthy volunteers were recruited as reported previously (21, 22). Following the collection of overnight fasted blood samples all the subjects underwent a 5-h saline infusion with insulin sensitivity (IS) assessed by a hyperinsulinemic-euglycemic clamp (HIEC) in the final 2 h to determine the glucose disposal rate (M value). A week later, all participants underwent 5 h intralipid infusion (20% soybean oil, 1.2% egg yolk phospholipids, and 2.2% glycerol; Kabi Fresenius Pharmacia) with a HIEC in the last 2 h to determine the *M*-value (21). HIEC was started using intravenous soluble insulin at a rate of 80 mU/m^2^ surface area/min for the first 20 min, followed by a constant rate of 40 mU/m^2^ surface area/minute for the remaining 100 min. Plasma glucose was clamped at 5.0 mmol/L with a variable infusion rate of 20% dextrose, adjusted relative to arterialized blood glucose measurements undertaken every 5 min. Blood samples were collected at 0, 180, 240, and 300 min for measurement of various proteins. On completion of the procedure all the participants underwent supervised moderate intensity (60% maximum oxygen consumption) exercise, 3 h weekly for 8 weeks. Healthy control women had the initial clamp in the first week of their menstrual cycle, whilst PCOS women were clamped after 6 weeks amenorrhea as reported previously (21).

### Exercise Intervention

All the participants underwent a 1-h supervised walking exercise program three times per week for a total of 8 weeks in the department of Sports, Health and Exercise Science, University of Hull. VO_2_ max was measured in a motorized treadmill with warm-up followed by speeding to walking pace and increasing the walking pace every minute until the study participants could not keep pace with the treadmill or become too tired to continue. Exercise sessions achieved heart rate equivalent to 60% of baseline VO_2_ max. The heart rate and inspired/expired gas fractions were monitored continuously using the same instrument used in the training program for each patient (16, 23). After the exercise intervention, anthropometric measurements, blood biochemistry, saline, lipid infusions, and HIEC were repeated in these subjects as reported previously (22). Repeat saline studies for each individual were performed immediately after the 3-month interventional exercise phase and the lipid studies performed 1 week later as for the initial saline lipid sequence.

### Biochemical Measurements

Plasma levels of FGF19 were measured using quantitative sandwich ELISA kit (R&D Systems, MN, USA; Catalog# DF1900). The kit had a mean minimum detectable dose (MDD) of 1.17 pg/ml, quantification range of 15.6–1,000 pg/ml, intra-assay coefficient of variation (CV) <6.4% and inter-assay CV <5.5%. Fetuin A concentrations in serum samples were quantified using a commercially available Quantikine ELISA kit (R&D Systems, MN, USA; Catalog# DFTA00). The kit has a mean MDD of 0.62 ng/ml, quantification range of 7.8–500 ng/ml, inter-assay CV <7.4% and intra-assay CV <8.4%. Serum concentrations of FGF21 and FGF23 were measured using Milliplex MAP kit -Human Liver Protein Magnetic Bead Panel (EMD Millipore, MA, USA, Catalog # HLPPMAG-57K). The kit had a detection range of 0.01–10 ng/mL for FGF21(inter-assay CV <12% and intra-assay CV <2%) and 0.04–30 ng/mL for FGF23 (inter-assay CV <15% and intra-assay CV <3%). Below mentioned variables were measured as reported previously (21) serum insulin was measured using a competitive chemiluminescent immunoassay (Euro/DPC, Llanberis, UK). Plasma glucose was measured by Synchon LX 20 analyzer (Bechman-Coulter, High Wycombe, UK). Triglycerides (TG) and total cholesterol measurements were done using a Synchon LX 20 analyzer (Beckman-Coulter). The free androgen index (FAI) was calculated by dividing the total testosterone by SHBG, and then multiplying by 100. Serum testosterone (nmol/L) was assessed by high-performance liquid chromatography linked to tandem mass spectrometry (Waters Corporation, Manchester, UK), sex hormone binding globulin (SHBG) (nmol/L) levels were measured by immunometric assay with fluorescence detection on the DPC Immulite 2,000 analyzer (Euro/DPC, Llanberis UK). FSH (iu/L) was measured by an Architect analyzer (Abbott Laboratories, Maidenhead, UK); TCH (mmol/L), TG (mmol/L), and HDL (mmol/L) were measured using a Synchron LX 20 analyzer (Beckman-Coulter); LDL (mmol/L) was calculated using the Friedewald equation. Plasma glucose was measured using a Synchron LX 20 analyzer (Beckman-Coulter). NEFA was measured using an enzymatic colorimetric method (Wako NEFA-H2) on a Konelab20 auto analyzer with a coefficient of variation of 1.4%. All the above measurements were performed according to manufacturer's recommended protocol.

### Statistical Analysis

All measurements are expressed as means ± standard deviation (SD). The differences in baseline demographic, clinical, and biochemical data between PCOS vs. control were assessed by unpaired *t*-test. Comparisons of FGF19, FGF21, FGF23, and fetuin-A in the control and PCOS groups before and after the exercise intervention were determined by two-way ANOVA. For saline, intralipid, and insulin infusion experiments repeated measure one-way ANOVA analysis was performed to determine significant differences between all the timepoints within control group and PCOS separately. A *post hoc* analysis (Bonferroni) correction was performed to evaluate significant differences between each time point to its respective baseline measurements for control and PCOS groups that were done separately. Comparison between control and PCOS groups during saline-insulin infusion and lipid-insulin infusion studies were done by two-way RM ANOVA analysis. Pearson Rank correlation was used to evaluate significant associations between FGF19, FGF21, FGF23, and fetuin-A with clinical variables. Statistical significance was set at *p* < 0.05. The statistical package SPSS 22.0 and Graphpad prism 5 software was used for the data analysis and graphical representation of results.

## Results

### Characteristics of the Study Subjects

**Figure 1:**
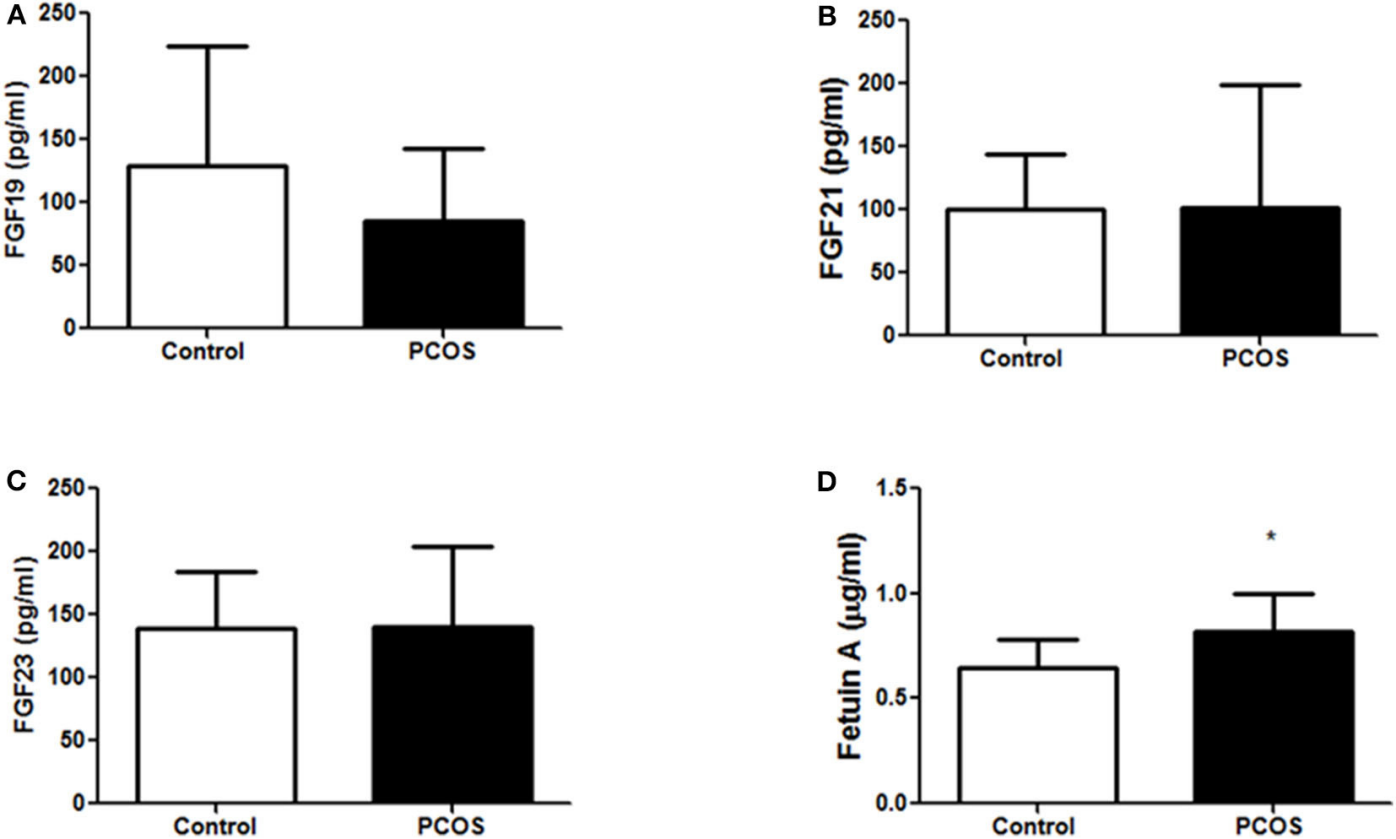
Baseline expression of metabolic hormones **(A)** FGF19, **(B)** FGF21, **(C)** FGF23, and **(D)** fetuin-A in control (*n* = 10) and PCOS (*n* = 11) women. The un-paired *t*-test was performed to compare differences between control and PCOS group. A *p < 0.05 of was considered significant. FGF19, fibroblast growth factor 19; FGF21, fibroblast growth factor 21; FGF23, fibroblast growth factor 23.

Demographics, clinical variables, and biochemical measurements of the study participants were reported previously (21). In brief, women with PCOS had increased body weight, higher insulin resistance, and elevated liver enzymes but decreased sex hormone binding globulin (Supplementary Table 1). At baseline, plasma concentrations of fetuin-A were elevated in PCOS (*P* < 0.05) but FGF19, FGF21, FGF23 levels were similar in the PCOS and control groups (Figure 1). Following exercise there was a reduction in waist circumference (*p* = 0.05), but no significant changes in weight (*p* = 0.40) (21). Unpaired *t*-test was performed to determine significant differences between control and PCOS baseline measurements.

### Effects of Acute Insulin Infusion on Circulating Lipids, Endocrine FGFs, and Fetuin-A

As reported previously (21), insulin sensitivity index (*M*-value) was lower in PCOS (*P* < 0.05); and insulin infusion suppressed plasma concentrations of TG (*P* < 0.0001) and non-esterified fatty acids (NEFA) (*P* < 0.0001). Two-way RM ANOVA analysis comparing between control and PCOS group showed no changes for FGF19, FGF21, FGF23, and Fetuin-A between the groups. One-way ANOVA analysis comparing differences for each of FGF19, 21, 23, and fetuin-A at the 3 time points of baseline, 180 and 300 min within the control group or within the PCOS groups showed that insulin infusion significantly increased the plasma concentrations of FGF21 (*P* < 0.05) in both PCOS and control women by 77 and 63%, respectively (*P* < 0.01); decreased FGF19 was seen only in the control group as assessed by one-way ANOVA comparing all the timepoints within control group, and there were no effects on plasma levels of FGF23 or fetuin-A in either groups (Table 1). Further *post hoc* analysis showed compared to baseline (0 min), insulin infusion at 300 min reduced FGF19 in controls. In contrast, insulin increased FGF21 secretion at 300 min both in control and PCOS compared to their respective baseline (0 min) measurements in the *post hoc* analysis.

**Table 1:** Effects of acute insulin administration on circulating lipids, endocrine FGFs, and fetuin-A in control (*n* = 10) and PCOS (*n* = 11) subjects.

		**Control**	**One-way**	***post-hoc***	**PCOS**	**One-way**	***post-hoc***	**Two-way**
		**(*N* = 10)**	**ANOVA**	**analysis**	**(*N* = 11)**	**ANOVA**	**analysis**	**RM ANOVA**
	**Infusion (min)**	**Mean ± SD**	***p***	***p***	**Mean ± SD**	***p***	***p***	***p***
FGF19 (pg/ml)	Baseline (0)	109.30 ± 52.2	*		83.32 ± 50.8	NS		NS
	Saline (180)	85.37 ± 32.2			61.59 ± 39.6			
	Saline + Ins (300)	63.39 ± 30.9		^#^	66.37 ± 50.5			
FGF21 (pg/ml)	Baseline (0)	120.00 ± 87.7	*		167.00 ± 89.2	^**^		NS
	Saline (180)	121.50 ± 91.7			128.50 ± 58.1			
	Saline + Ins (300)	215.00 ± 55.5		^#^	208.00 ± 103.7		^##^	
FGF23 (pg/ml)	Baseline (0)	120.00 ± 43.5	NS		125.6 ± 45.0	NS		NS
	Saline (180)	88.33 ± 41.7			96.67 ± 23.5			
	Saline + Ins (300)	86.67 ± 29.4			103.30 ± 24.5			
Fetuin-A (μg/ml)	Baseline (0)	0.72 ± 0.2	NS		0.82 ± 0.2	NS		NS
	Saline (180)	0.72 ± 0.1			0.80 ± 0.2			
	Saline + Ins (300)	0.70 ± 0.2			0.78 ± 0.1			
*M*-value		5.0 ± 2.0			3.3 ± 0.8			

One-way ANOVA group analysis was performed in controls to determine significant differences for FGF19, 21, 23 and fetuin-A between time points within control group

*p < 0.05. post hoc analysis with Bonferroni correction was performed for subgroup analysis to compare each time point measurements within control group to control baseline expression

##p < 0.001;

#p < 0.05.

This one-way ANOVA group analysis was repeated in PCOS women separately

**p < 0.001, and post hoc analysis was also repeated comparing each time point measurements within PCOS group to baseline expression levels in the PCOS group

*^##^p < 0.001. Two-way RM ANOVA analysis was performed to compare between control and PCOS group. FGF19, fibroblast growth factor 19; FGF21, fibroblast growth factor 21; FGF23, fibroblast growth factor 23; M-value, Insulin sensitivity index; NS, non-significant*.

### Effects of Intralipid and Combination of Intralipid and Insulin Infusions on Circulating Endocrine FGFs and Fetuin-A

As reported previously, intralipid infusion dramatically increased plasma TG, and NEFA concentrations in control and PCOS women; and had no effects on cholesterol levels (15). Two-way RM ANOVA analysis comparing between control and PCOS showed a significant increase in FGF21 (*P* < 0.05) between the groups and no changes were observed for FGF19, FGF23, and Fetuin-A. However, one-way ANOVA analysis assessing changes within the control or the PCOS groups during the time course showed that intralipid and a combination of intralipid and insulin suppressed FGF19 in control (*P* < 0.01) and in the PCOS groups (*P* < 0.0001). Further *post hoc* analysis showed that the intralipid infusion suppressed the plasma concentration of FGF19 in both control (*P* < 0.01) and PCOS (*P* < 0.0001) to 50 and 39% of baseline at 60 min, respectively; and the levels remained suppressed during insulin infusion at 300 min (Table 2). In contrast, one-way ANOVA showed both intralipid and combination of intralipid and insulin increased FGF21 both in control (*P* < 0.0001) and PCOS group (*P* < 0.0001) over the time course. Further *post hoc* analysis showed that intralipid infusion in control subjects had a biphasic effect on FGF21; decreased FGF21 concentrations to 70% of baseline at 60 min but increased its concentrations to 141% of baseline at 180 min (*P* < 0.05, Table 2). A similar pattern was also observed in PCOS women (*P* < 0.01, Table 2). Insulin infusion, added to intralipid during the last 2 h, further increased plasma FGF21 to 247% and 585% of baseline at 240 and 300 min, respectively, in control subjects (*P* < 0.0001, Table 2, *post-hoc* analysis). A similar pattern was also observed in PCOS women (*P* < 0.0001, *post-hoc* analysis Table 2). Intralipid infusion alone had no effect on FGF23 or fetuin-A in both groups (Table 2); the addition of insulin along with intralipid decreased FGF23 only in the control group (*P* < 0.01). And did not change the plasma levels of FGF19 or fetuin-A during the euglycemic clamp in PCOS women (Table 2).

**Table 2:** Effects of acute intralipid and combination of intralipid plus insulin administration on circulating lipids, endocrine FGFs, and fetuin- in control (*n* = 10) and PCOS (*n* = 11) subjects.

		**Control**	**One-way**	***post-hoc***	**PCOS**	**One-way**	***post-hoc***	**Two-way**
		**(*N* = 10)**	**ANOVA**	**analysis**	**(*N* = 11)**	**ANOVA**	**analysis**	**RM ANOVA**
	**Infusion (min)**	**Mean ± SD**	***p***	***p***	**Mean ± SD**	***p***	***p***	***p***
FGF19 (pg/ml)	Baseline (0)	129.5 ± 94.3	**		84.67 ± 58.4	***		NS
	Lipid (60)	65.08 ± 36.0		#	33.4 ± 15.8		###	
	Lipid (180)	53.1 ± 37.3		##	35.73 ± 18.9		###	
	Lipid + Ins (240)	44.07 ± 24.7		##	41.15 ± 19.8		##	
	Lipid + Ins (300)	57.10 ± 19.5		#	45.79 ± 19.9		##	
FGF21 (pg/ml)	Baseline (0)	163.00 ± 102.0	***		143.60 ± 124.6	***		$
	Lipid (60)	114.50 ± 91.2			138.20 ± 174.6			
	Lipid (180)	231.00 ± 164.8		###	309.10 ± 174.8		###	
	Lipid + Ins (240)	403.00 ± 275.7		###	600.90 ± 339.6		###	
	Lipid + Ins (300)	959.00 ± 618.3		###	1056.00 ± 386.5		###	
FGF23 (pg/ml)	Baseline (0)	139.00 ± 45.3	**		138.20 ± 62.9	NS		NS
	Lipid (60)	105.00 ± 47.2			136.40 ± 62.49			
	Lipid (180)	103.00 ± 55.8			123.60 ± 58.01			
	Lipid + Ins (240)	85.00 ± 26.7		##	120.00 ± 59.8			
	Lipid + Ins (300)	116.00 ± 57.4			159.10 ±109.2			
Fetuin-A (μg/ml)	Baseline (0)	0.68 ± 0.2	NS		0.82 ± 0.2	NS		NS
	Lipid (60)	0.74 ± 0.2			0.84 ± 0.2			
	Lipid (180)	0.71 ± 0.1			0.78 ± 0.2			
	Lipid + Ins (240)	0.68 ± 0.1			0.80 ± 0.2			
	Lipid + Ins (300)	0.72 ± 0.1			0.81 ± 0.2			
*M*-value		2.4 ± 1.7			1.2 ± 0.8			

One-way ANOVA group analysis was performed in controls to determine significant differences between time points for FGF19, 21, 23 and fetuin-A within control group

***p < 0.0001;

**p < 0.001. post hoc analysis with Bonferroni correction for subgroup analysis comparing each the time point with control baseline expression

###p < 0.0001;

##p < 0.001;

#p < 0.05. Similarly, One-way ANOVA group analysis was performed in PCOS women separately

*** p < 0.0001;

**p < 0.001, with the post hoc analysis undertaken to compare each time point measurements within the PCOS group to baseline expression levels in the PCOS group ###p < 0.0001; ##p < 0.001. Two-way RM ANOVA analysis was performed to compare between control and PCOS group; ^$^p < 0.05. FGF19, fibroblast growth factor 19; FGF21, fibroblast growth factor 21; FGF23, fibroblast growth factor 23; M-value, Insulin sensitivity index; NS, non-significant.

### Moderate Exercise Training Does Not Alter Circulating Endocrine FGFs and Fetuin-A

**Figure 2:**
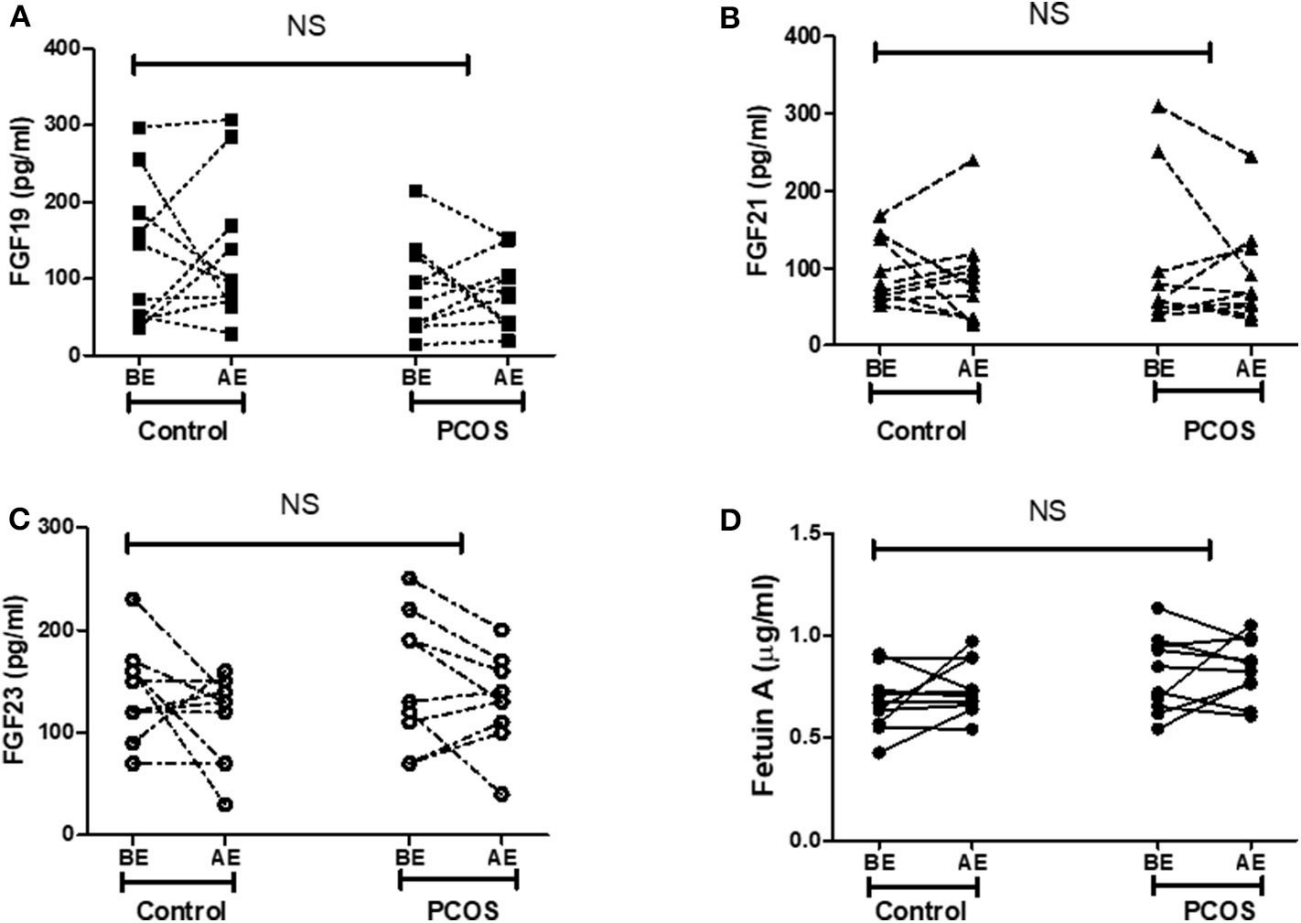
Effect of exercise on expression of metabolic hormones **(A)** FGF19, **(B)** FGF21, **(C)** FGF23, and **(D)** fetuin-A in control (*n* = 10) and PCOS (*n* = 10) women. BE, before exercise; AE, after exercise. Statistical differences in metabolic hormones expression following exercise in control and PCOS were measured by two-way ANOVA. FGF19, fibroblast growth factor 19; FGF21, fibroblast growth factor 21; FGF23, fibroblast growth factor 23; NS, non-significant.

As reported previously, exercise improved insulin sensitivity and overall fitness in both control and PCOS women with an improvement of VO2 max. However, there were no significant changes in the plasma levels of metabolic hormones FGF19, FGF21, FGF23, and fetuin-A in either group before or after exercise (Figure 2). Comparison of FGF19, FGF21, FGF23, and fetuin-A in the control and PCOS group before and after the exercise intervention was determined by two-way ANOVA.

### Correlation Analysis of FGF19, FGF21, FGF23, and Fetuin-A With Covariates

In all the participants, Pearson Rank analysis was performed to study the association of endocrine FGFs and fetuin-A with baseline clinical and biochemical parameters (Table 3). Our analysis showed that plasma FGF19 levels significantly correlated negatively with hip circumference (*r* = −0.433, *P* < 0.05). FGF-21 correlated positively with plasma FGF-23 (*r* = 0.456, *P* < 0.05) and diastolic blood pressure (*r* = 0.487, *P* < 0.05). FGF-23 showed negative association with total cholesterol concentration (*r* = −0.478, *P* < 0.05). Similarly, fetuin-A correlated positively with weight (*r* = 0.473, *P* < 0.05), BMI (*r* = 0.521, *P* < 0.05), hip circumference (*r* = 0.454, *P* < 0.05), plasma glucose (*r* = 0.478, *P* < 0.05), and insulin (*r* = 0.571, *P* < 0.01) levels, and HOMA-IR (*r* = 0.616, *P* < 0.01).

**Table 3:** Pearson Rank correlation of FGF19, FGF21, FGF23, and fetuin-A with anthropometric and blood biochemical variables.

	**FGF-19**		**FGF-21**		**FGF-23**		**Fetuin-A**	
Pearson correlation	*r*	*p*	*r*	*p*	*r*	*p*	*r*	*p*
FGF-19	1.000		0.079	0.741	0.301	0.185	−0.016	0.944
FGF-21	0.079	0.741	1.000		0.456*	0.043	−0.152	0.521
FGF-23	0.301	0.185	0.456*	0.043	1.000		−0.089	0.700
Fetuin-A	−0.016	0.944	−0.152	0.521	−0.089	0.700	1.000	
age	−0.035	0.879	0.118	0.621	−0.196	0.395	0.319	0.159
Height	−0.224	0.329	0.110	0.646	0.186	0.419	0.016	0.945
Weight	−0.278	0.223	−0.188	0.427	0.128	0.581	0.473*	0.030
BMI	−0.213	0.353	−0.227	0.336	0.065	0.780	0.521*	0.015
Waist	−0.295	0.195	−0.144	0.543	0.031	0.893	0.430	0.052
Hip	−0.433*	0.050	−0.103	0.665	−0.009	0.969	0.454*	0.039
WHR	0.128	0.580	−0.158	0.506	0.127	0.582	0.138	0.551
SBP	−0.013	0.954	0.340	0.143	0.057	0.806	0.183	0.428
DBP	−0.367	0.102	0.487*	0.030	0.272	0.233	0.383	0.087
PG	0.296	0.193	−0.158	0.505	−0.252	0.270	0.478*	0.028
Insulin	0.019	0.935	−0.178	0.453	0.249	0.277	0.571**	0.007
HOMA-IR	0.059	0.798	−0.185	0.436	0.171	0.460	0.616**	0.003
M-value	0.248	0.279	−0.158	0.505	−0.240	0.295	−0.303	0.182
NEFA	−0.138	0.550	−0.056	0.813	−0.425	0.055	−0.257	0.261
TCH	−0.291	0.200	0.009	0.971	−0.478*	0.028	−0.176	0.444
Testosterone	0.071	0.759	−0.250	0.288	0.536*	0.012	0.231	0.314
FAI	−0.190	0.408	−0.410	0.072	0.222	0.334	0.375	0.094
SHBG	0.406	0.068	0.487*	0.030	0.008	0.971	−0.286	0.210
LH	−0.248	0.292	0.182	0.456	0.264	0.261	0.029	0.902
FSH	0.157	0.509	0.036	0.883	−0.208	0.379	0.445*	0.049
Estradiol	−0.297	0.203	−0.082	0.739	0.364	0.114	−0.081	0.735
TG	−0.240	0.295	0.054	0.820	−0.220	0.338	0.010	0.967
HDL	−0.193	0.403	−0.170	0.473	−0.487*	0.025	−0.296	0.192
LDL	−0.164	0.478	0.212	0.370	0.275	0.228	0.024	0.919
ALT	−0.190	0.423	−0.266	0.271	0.010	0.968	0.235	0.319
HbA1c	−0.153	0.532	−0.047	0.854	−0.296	0.219	0.339	0.155
Prolactin	0.272	0.259	−0.309	0.212	0.122	0.620	−0.162	0.507
TSH	−0.029	0.902	−0.015	0.949	0.163	0.479	−0.079	0.732
DHEAS	0.130	0.596	−0.184	0.466	0.311	0.194	−0.381	0.108
Androsterone	−0.162	0.508	−0.346	0.159	0.235	0.332	−0.283	0.241

*p is the two tailed significance and r is Pearson correlation coefficient. FGF19, fibroblast growth factor 19; FGF21, fibroblast growth factor 21; FGF23, fibroblast growth factor 23; BMI, Body mass index; WHR, Waist to hip ratio; SBP, systolic blood pressure; DBP, diastolic blood pressure; PG, Plasma glucose; HOMA-IR, Homeostatic Model Assessment of Insulin Resistance; M-value, Insulin sensitivity index; NEFA, non-esterified fatty acid; TCH, Total cholesterol; FAI, Free androgen index; SHBG, Sex hormone-binding globulin; LH, Luteinizing hormone; FSH, Follicle stimulating hormone; TG, Triglycerides; HDL, High density lipoprotein; LDL, Low density lipoprotein; ALT, Alanine transaminase; HbA1c, hemoglobin A1c; TSH, Thyroid stimulating hormone; DHEAS, Dehydroepiandrosterone*.

## Discussion

Endocrine FGFs and fetuin-A regulate a broad spectrum of metabolic functions such as glucose, lipid metabolism, and energy homeostasis by regulating several signaling cascades that lead to altered insulin sensitivity (11, 24). We hypothesized that disrupting glucose and lipid homeostasis by acute intralipid and insulin administration or via moderate exercise may differentially modulate circulating endocrine FGFs and fetuin-A in healthy women and women with PCOS. Thus, to address this hypothesis, we analyzed plasma levels of endocrine FGFs and fetuin-A before and after a supervised exercise program. We further studied the role of short-term hyperlipidemia mediated insulin resistance induced by intravenous administration of intralipid via lipid clamp and insulin administration via HIEC on plasma levels of endocrine FGFs and fetuin-A. As reported previously, intralipid infusion induced insulin resistance both in control and PCOS subjects as shown by the lowering of insulin stimulated glucose disposal rates during the intralipid clamp (16). Compared to controls, the insulin stimulated glucose disposal rate showed greater suppression in PCOS (16).

In our study, fetuin-A was found to be significantly higher in PCOS compared to controls, but there were no significant differences observed for FGF19, FGF21, and FGF23 between the groups. Plasma fetuin-A levels positively associated with risk factors for IR such as weight, BMI, plasma glucose, insulin, and HOMA-IR. In concordance with our finding in PCOS, fetuin-A levels were reported to be elevated in insulin-resistant T2D subjects, positively correlated with insulin resistance markers such as HOMA-IR, glycated hemoglobin, low-density lipoprotein cholesterol, BMI, and negatively correlated with fasting insulin, high-density lipoprotein cholesterol, and HOMA-β-cell insulin secretion index (25), and fetuin-A was shown to antagonize insulin action at cellular levels with direct inhibition of insulin binding to its receptor (26, 27). Evidence from a cellular study using pancreatic-β cells (MIN6) suggested fetuin-A to be a key intermediate protein in lipid-induced cell death (28); however, we did not observe any changes in fetuin-A levels following intralipid administration in our study subjects.

The metabolic effects of FGF19 and FGF21 are broadly similar and both regulate glucose homeostasis, improve insulin sensitivity, and lower body weight (29). However, there are subtle differences with regards to lipid metabolism. FGF19 has a dual action on lipid metabolism; short term treatment with FGF19 in diet induced obese mice resulted in a transient rise in plasma TG by day 4 that returned to near baseline levels by 10 days. On the other hand, long term treatment with FGF19 lowers plasma TG and cholesterol in the same mouse model. The effects of FGF19 are slightly different in leptin-deficient obese ob/ob mice where both 1–2-week treatment with FGF19 resulted in a consistent rise in TG and cholesterol levels (30). Despite this rise in plasma lipids, FGF19 administration increased insulin sensitivity evidenced by lowering plasma glucose and insulin (30). Transgenic mice over-expressing the FGF19 protein are resistant to diet-induced diabetes. These animal observations taken together indicates that pharmacological administration and transgenic expression of FGF19 increase insulin sensitivity.

Interestingly, FGF19 levels were negatively correlated with fasting plasma glucose (31) and shown to be decreased in impaired fasting glucose, obesity, T2D, and NAFLD subjects. This suggest a physiological role for endogenous FGF19 in glucose metabolism and insulin sensitivity. We also observed lower plasma FGF19 levels in insulin-resistant women with PCOS; however, this was not statistically significant possibly due to our smaller sample size. Available evidence in the literature, together with our findings support the hypothesis that FGF19 is impaired in subjects with IR.

In our study, insulin infusion increased FGF21 but did not alter FGF19, FGF23, and fetuin-A in both PCOS and controls; this is in agreement with previous reports that showed a rise in serum and skeletal muscle FGF21 levels in overweight, obese, and T2D subjects following an insulin infusion, (32) and no change in FGF19 (33) and FGF23 (34) during hyperinsulinemia in healthy volunteers. The increase in FGF21 by intralipid infusion observed in our study may be a compensatory mechanism to counter the dysregulated insulin signaling induced by intralipid and the insulin clamp, and this raised FGF21 may contribute to the regulation of glucose uptake by muscle and adipose tissue, thus regulating glucose homeostasis. In agreement with this hypothesis data in rodents suggest that overproduction of FGF21 prevents age-associated IR (35) and improves metabolic abnormalities.

It is well-recognized that an intralipid infusion induces IR through the Randle cycle by increasing plasma FFA and triglycerides (16, 36). The elevated FFA further promotes release of proinflammatory cytokines, alters carbohydrate metabolism, induces IR in skeletal and cardiac muscle (37) and thus triggers peripheral IR in healthy subjects. These effects could be amplified in women with PCOS who have a higher degree of pre-existing IR. In our study, lipid infusion stimulated steep rises in FGF21, suppressed FGF19 and did not alter FGF23 and fetuin-A levels. This is in agreement with a previous study that also reported increased plasma FGF21 and no changes in fetuin-A following an intralipid infusion in healthy volunteers (38). Similar findings were also observed in dairy cows with increased NEFA, plasma, and hepatic FGF21 production increased following intralipid administration (39). Further in our study groups, the addition of insulin along with lipid did not alter fetuin-A levels and did not reverse lipid mediated suppression of FGF19. Interestingly, a further rise in FGF21 was observed both in control and PCOS women following administration of insulin along with intralipids suggesting the synergistic action of intralipid and insulin on FGF21. In controls, FGF23 levels were transiently suppressed following 60 min of insulin along with intralipids and returned to near baseline values by 120 min. Of relevance, insulin-mediated suppression of FGF23 has been previously reported in cell culture models (osteoblast-like cells UMR106 cells) and in rodents. In rodent's, insulin deficiency increased serum FGF23 which was reversed by insulin administration; and a strong negative association was observed between FGF23 and insulin in humans (40).

Regular physical activity promotes many health benefits and reduces the risk of insulin resistance (19). Despite this, studies have shown endurance training does not offer any protection from FFA induced insulin resistance in trained individuals (17). Disparity in the literature exists for effects of exercise on endocrine FGFs and fetuin-A. In healthy lean men, 60-min short term high intensity endurance exercise did not alter FGF19 levels, although resistance exercise reduced FGF19 significantly (41). In the same study, FGF21 increased with endurance but not resistance exercise. (41) in healthy lean but not overweight, obese and T2D subjects (32). Similarly, effects of exercise on FGF23 and fetuin-A are also contradicting in mice, both acute and chronic exercise increased serum FGF23 levels and was associated with an increase in exercise performance (42). However, in human volunteers, exercise did not alter circulating FGF23 (43). In elderly over-weight men, 6 months of aerobic exercise increased circulating fetuin-A. This raised fetuin-A was not related to insulin sensitivity but was associated with VO2max (44). In a recently published systematic review and meta-analysis study in obese, T2D undergoing moderate to intense supervised exercise reported a reduction in fetuin-A following exercise (45). In our study cohort, 8 weeks of moderate intensity exercise did not have any significant effects on FGF19, FGF21, FGF23, and fetuin-A levels. Our results on FGF21 are in agreement with studies from Kruse et al. (32) where the authors studied effects of 10-weeks of aerobic training on FGF21 levels in serum samples from lean, obese, and T2D subjects and found no significant changes in FGF21 levels following exercise. Our results are in contrast to that reported by Morville et al. (41) who reported whilst FGF19 was unchanged FGF21 increased with cycling exercise in trained males; however, our study was conducted in over weight PCOS and control women and the duration of exercise interval was more chronic in comparison. Fetuin-A was found to be reduced following exercise in adults and elderly (45), we did not observe any changes in fetuin-A following exercise this may possibly due to our smaller sample size. Despite not having any major impact on endocrine FGFs or fetuin, exercise improved cardio-respiratory fitness (Vo2 max) and insulin sensitivity measured by changes in HOMA-IR, glucose disposal rate and *M*-value (16). This is in agreement with previous studies which have also reported no significant changes in body weight following regular exercise (46). In spite of not having impact on weight loss exercise reduces cardiometabolic risk, triglycerides, IR (47), regularizing hormonal problems, improve insulin sensitivity, improve ovulation (48) and has better outcome on fertility rates in PCOS women.

This study has number of limitations. The study was conducted in a small group of PCOS and healthy women given the intense nature of study protocol requiring frequent blood sampling, such interventional studies are challenging to perform in a large group of subjects; hence our findings may not be generalized. Similarly, our study did not take into account ethnicity (all subjects were Caucasian), metabolic abnormalities, phenotypic differences that usually exists in PCOS women. In addition, BMI stratification would need to be undertaken in future studies that would affect insulin resistance and hence may also influence expression of the proteins measured in this study. Because of the small population selected it is possible that some of the protein changes may have been missed due to the power of the study.

## Conclusion

This is the first study reporting the effects of intralipid and insulin on endocrine FGFs and fetuin-A in women with PCOS. Overall, these data suggest that FGF19 and FGF21 may have a modulatory role in lipid and insulin metabolism as shown by hyperinsulinemia and the lipid infusion. Lipid and insulin synergistically increased FGF21 in healthy and insulin resistant women with PCOS; hyperinsulinemia suppressed FGF19 in controls (Table 2). However, lipid infusion mediated suppression of FGF19 was not responsive to insulin in both controls and in women with PCOS. Neither insulin nor intralipid- induced any significant changes in the expression of FGF23 and fetuin-A.

The ISRCTN number for this study is ISRCTN42448814.

## Data Availability Statement

The raw data supporting the conclusions of this article will be made available by the authors, without undue reservation.

## Ethics Statement

All patients provided written informed consent to participate in the study, which was conducted in accordance with the Declaration of Helsinki and Good Clinical Practice. This study protocol was approved by the Leeds Central Research Ethics Committee, Yorkshire and Humber.

## Author Contributions

MR, IB, JJ, MB, and TAS performed the measurements and contributed to manuscript. MR wrote the manuscript. SA, TS, MMA recruited subjects and involved in sample collection and data analysis. TAS, KS, and MA researched the data and contributed to manuscript. MR, A-BA-S, MS, and SA conceptualized the study designed the experiments, supervised progress, analyzed data, and approved the final version of the article. All the authors reviewed and revised the manuscript.

## Conflict of Interest

The authors declare that the research was conducted in the absence of any commercial or financial relationships that could be construed as a potential conflict of interest.

## Supplementary Material

Supplementary Table 1 Baseline anthropometric, biochemical and hormonal measurements in the study subjects.

## Abbreviations

PCOS: Polycystic ovary syndrome
T2D: Type 2 diabetes
IR: insulin resistance
IS: Insulin sensitivity
Endocrine FGF: FGF19, FGF21 and FGF23
BMI: body mass index
SBP: systolic blood pressure
DBP: diastolic blood pressure
TCH: total cholesterol
HDL: high density lipoprotein
LDL: low density lipoprotein
TG: triglycerides
HbA1c: glycated hemoglobin
ALT: alanine aminotransferase
HIEC: hyperinsulinemic-euglycemic clamp
FGF: fibroblast growth factor
NAFLD: non-alcoholic fatty liver disease
HOMA-IR: homeostatic model assessment of insulin resistance
NEFA: non-esterified fatty acids
FSH: follicle-stimulating hormone
SHGB: sex hormone binding globulin
FAI: free androgen index.
