# Supplementary material for: Dynamic Changes in Circulating Endocrine FGF19 Subfamily and Fetuin-A in Response to Intralipid and Insulin Infusions in Healthy and PCOS Women
Source: Front Endocrinol (Lausanne). 2020 Sep 30;11:568500. doi: 10.3389/fendo.2020.568500 (PMC7554576; doi:10.3389/fendo.2020.568500)
Supplement: Supplementary Table 1 — Baseline anthropometric, biochemical and hormonal measurements in the study subjects. [file Table_1.docx]

Supplementary Table 1.

Baseline anthropometric, biochemical and hormonal measurements in the study subjects

|  | Control (N=10) Mean **±** S.D | PCOS (N=11) Mean **±** S.D | p |
| --- | --- | --- | --- |
| Age (years) | 24.3 **±** 6.2 | 28.0 **±** 6.7 | NS |
| BMI (kg/m^2^) | 25.9 **±** 5.6 | 30.1 **±** 5.8 | NS |
| WHR (cm) | 0.8 **±** 0.1 | 0.8 **±** 0.1 | NS |
| SBP (mmHg) | 115.0 **±** 9.4 | 120.0 **±** 10.5 | NS |
| DBP (mmHg) | 72.6 **±** 9.5 | 76.6 **±** 7.5 | NS |
| PG (nmol/L) | 4.8 **±** 0.5 | 4.7 **±** 0.5 | NS |
| HbA1c (mmol/mol) | 33.4 **±** 6.4 | 33.9 **±** 2.8 | NS |
| Insulin (pmol/L) | 45.0 **±** 22.7 | 82.9 **±** 49.0 | P<0.05 |
| HOMA-IR | 1.6 **±** 0.9 | 3.24 **±**1.9 | P<0.05 |
| NEFA (µmol/L) | 546.1 **±** 222.5 | 557.7 **±** 218.8 | NS |
| TCH (mmol/L) | 4.6 **±** 0.8 | 4.1 **±** 0.7 | NS |
| TG (mmol/L) | 0.9 **±** 0.2 | 1.2 **±** 0.5 | NS |
| HDL (mmol/L) | 1.5 **±** 0.5 | 1.3 **±** 0.4 | NS |
| LDL (mmol/L) | 2.7 **±** 0.6 | 2.3 **±** 0.5 | NS |
| Testosterone (nmol/L) | 1.1 **±** 0.4 | 1.3 **±** 0.6 | NS |
| SHBG (nmol/L) | 69.6 **±** 29.6 | 25.8 **±** 18.5 | P<0.001 |
| FAI | 2.2 **±** 2.1 | 6.6 **±** 3.1 | P<0.001 |
| LH (iU/L) | 7.3 **±** 12.8 | 5.0 **±** 4.1 | NS |
| FSH (iU/L) | 5.0 **±** 1.9 | 6.7 **±** 11.5 | NS |
| Estradiol (pmol/L) | 179.4 **±** 203.6 | 180.6 **±** 140.3 | NS |
| Prolactin (mU/L) | 457.8 **±** 233.1 | 356.5 **±** 100.6 | NS |
| TSH (mU/L) | 1.9 **±** 0.9 | 1.6 **±** 0.5 | NS |
| DHEAS (µmol/L) | 7.9 **±** 2.8 | 6.0 **±** 3.7 | NS |
| Androstenedione (nmol/L) | 10.0 **±** 3.9 | 11.0 **±** 5.1 | NS |
| ALT (U/L) | 17.1 **±** 5.5 | 28.5 **±** 13.7 | P<0.05 |

The differences in baseline demographic, clinical and biochemical data between PCOS vs. control subjects were assessed by unpaired t-test. *p<0.05; **p<0.001; NS = non-significant. BMI = Body mass index; WHR = Waist to hip ratio; SBP = systolic blood pressure; DBP = diastolic blood pressure; PG = Plasma glucose; HbA1c = hemoglobin A1c; HOMA-IR = Homeostatic Model Assessment of Insulin Resistance; NEFA = [non-esterified](https://en.wiktionary.org/wiki/non-esterified_fatty_acid) fatty acid; TCH =Total cholesterol; TG = Triglycerides; HDL = High density lipoprotein; LDL = Low density lipoprotein; SHBG = Sex hormone-binding globulin; FAI = Free androgen index; LH = Luteinizing hormone; FSH = Follicle stimulating hormone; TSH = Thyroid stimulating hormone; DHEAS = Dehydroepiandrosterone; ALT = Alanine transaminase.
